# No difference in the functional improvements between unilateral and bilateral total knee replacements

**DOI:** 10.1186/s12891-018-2006-x

**Published:** 2018-03-21

**Authors:** Yu-Hao Huang, Chin Lin, Jia-Hwa Yang, Leou-Chyr Lin, Chih-Yuan Mou, Kwo-Tsao Chiang, Man-Gang Lee, Hsien-Feng Chang, Hsueh-Lu Chang, Wen Su, Shih-Jen Yeh, Hung Chang, Chih-Chien Wang, Sui-Lung Su

**Affiliations:** 1 School of Public Health, National Defense Medical Center, No.161, Min-Chun E. Rd., Sec. 6, Neihu, Taipei, 114 Taiwan, Republic of China; 2 Graduate Institute of Life Sciences, National Defense Medical Center, No.161, Min-Chun E. Rd., Sec. 6, Neihu, Taipei, 114 Taiwan, Republic of China; 3 Department of Orthopedics, Tri-Service General Hospital and National Defense Medical Center, No.325, Sec.2, Chenggong Rd., Neihu District, Taipei, 114 Taiwan, Republic of China; 4 Department of Aviation Medicine and Physical examination, National Defense Medical Center and Tri-Service General Hospital Songshan Branch, No.131, Jiankang Rd., Songshan District, Taipei, 10581 Taiwan, Republic of China; 5 Department of Surgery, Zuoying Branch of Kaohsiung Armed Forces General Hospital, No.553, Junxiao Rd., Zuoying Dist., Kaohsiung City, 813 Taiwan, Republic of China; 6 Department of Nursing, Tri-Service General Hospital, No.161, Min-Chun E. Rd., Sec. 6, Neihu, Taipei, 114 Taiwan, Republic of China; 7 Department of Research and Development, Da-Yeh University, No. 168, Xuefu Road, Dacun Township, Changhua County, 515 Taiwan, Republic of China; 8 Department of Physiology and Biophysics, National Defense Medical Center, No.325, Sec. 2, Chenggong Rd., Neihu District, Taipei City, 114 Taiwan, Republic of China; 9 Division of Thoracic Surgery, Tri-Service General Hospital, National Defense Medical Center, No.325, Sec. 2, Chenggong Rd., Neihu District, Taipei City, 114 Taiwan, Republic of China; 10 Graduate Institute of Medical Sciences, National Defense Medical Center, No.161, Min-Chun E. Rd., Sec. 6, Neihu, Taipei, 114 Taiwan, Republic of China

**Keywords:** Total knee replacement, Bilateral TKR, Unilateral TKR, Functional improvement, WOMAC, SF-36

## Abstract

**Background:**

Differences between staged bilateral total knee replacement (TKR) and simultaneous bilateral TKR have been investigated, but few studies have investigated differences in the functional improvements resulting from these methods. Therefore, this study investigates the different functional improvements between staged bilateral total knee TKR and simultaneous bilateral TKR.

**Methods:**

Among 144 potential bilateral TKR patients who were included in this study, 93 (64.6%) patients selected unilateral TKR and 51 (35.4%) selected bilateral TKR. Functional improvements were assessed using the Western Ontario and McMaster University osteoarthritis index (WOMAC) and the Medical Outcomes Trust Short Form-36 (SF-36), and patients were interviewed pre-operatively and after 6 months. A generalized equation was used to test for differences in functional improvements.

**Results:**

After TKR, pain, stiffness, function and total WOMAC scores were significantly reduced in both groups, with mean changes from − 26.6 to − 41.4 and from − 27.5 to − 42.2.The mean health change of SF-36 scores, physical component and mental component scores changed to 45.2 ± 18.2, 74.0 ± 15.4 and 77.0 ± 9.6, respectively, in Group 1 and 47.1 ± 17.1, 74.0 ± 15.2 and 75.5 ± 12.1, respectively, in Group 2.

Unilateral and simultaneous bilateral TKR produce similar functional improvements, although current work status may be a novel impact factor.

**Conclusion:**

No differences in functional improvements were identified between patients who selected unilateral versus bilateral TKR, indicating no recommendation for one procedure over the other.

**Electronic supplementary material:**

The online version of this article (10.1186/s12891-018-2006-x) contains supplementary material, which is available to authorized users.

## Background

Total knee replacement (TKR) is mainly offered to patients with end-stage osteoarthritis (OA) and has become more prevalent in recent years [1]. TKR is an effective intervention that improves quality of life, reduces pain and increases functional capability [2].

Many diagnoses of osteoarthritis (OA) are caused by aging, and the prevalence of bilateral symptomatic knees in these patients is 63.3% [3]. Patients with bilateral symptoms frequently require bilateral TKR, which can be performed as a one-stage simultaneous operation, or as a two-stage unilateral operation [4, 5]. Although patients are free to select the mode of TKR, it remains controversial which mode is better.

Previous studies have investigated differences in responses to staged bilateral and simultaneous bilateral TKR in terms of short-term discomfort [6], morbidity and mortality [7–10] and cost-effectiveness [11]. However, few studies have investigated differences in functional improvements. An Indian study described changes in functional improvements using the Western Ontario and McMaster University Osteoarthritis Index (WOMAC) in patients receiving simultaneous bilateral TKR [12], and another study from the United Kingdom reported changes in WOMAC scores in patients who received staged bilateral TKR [13]. However, these studies lacked suitable controls, precluding group comparisons between studies.

An Australian study compared the functional improvements between simultaneous bilateral and unilateral TKR [14], and this study reported that bilateral replacement patients reported better physical function and general health. However, the patients receiving bilateral and unilateral TKR in the above study had significant differences in the source of their health insurance [14], and this difference might cause a false result. The bilateral TKR group was younger and less likely to receive a pension. Instead, the group was more likely to have private health insurance, and most of them lived with others. Either of these situations may influence the patient’s costs over the post-operative year [14]. Taiwan had National Health Insurance (NHI), which almost covered the entire cost of TKR. This advantage might have reduced the economic inequalities and increased the homogeneity between patients receiving bilateral or unilateral TKR.

In summary, the available evidence is insufficient to explain with certainty the benefit of simultaneous bilateral TKR in functional outcomes. Thus, to inform clinical decisions, we investigated differences in functional improvements and assessed the potential impact factors between patients receiving unilateral and bilateral TKR.

## Methods

### Ethics statement and subject recruitment

The study was reviewed and approved by the institutional ethics committee. Written informed consent was obtained from all participants after thorough explanation of the study. Inclusion criteria were defined previously [15] and included (1) TKR surgery patients for the first time, (2) patients with bilateral knee OA and (3) patients with bilateral symptomatic knees. All patients were expected to eventually require bilateral TKR, and all TKRs were performed by a single surgeon. Moreover, patients choosing staged procedures needed more than 6 months of waiting time for the second surgery, and they followed the same post-operative recovery protocol. The waiting time included approximately 3 months for recovery and approximately 3 months for rehabilitation. Patients with serious co-morbidities (cancer, renal failure and infection) were excluded because of influences on functional measurements [16]. The above included criteria improved the homogeneity in the study population and might help to reduce potential confounding factors.

The patients who met the above criteria would attend an approximately half-hour explanation. The following information about the two TKR methods would be provided: (1) the waiting time in two-stage unilateral TKR, (2) higher anesthesia risks in simultaneous TKR [17], and (3) the short term discomfort in simultaneous TKR may be higher than in unilateral TKR [6]. In addition, physicians informed patients of the surgical options but did not influence patient decisions. Thus, modes of TKR were based on patient selection.

During the study period (from July 2009 to April 2010), a total of 169 TKR patients consented to participate in the study. Although 25 patients (16 patients who selected unilateral TKR and 9 patients who selected bilateral TKR) were lost to follow-up, no differences in the investigated characteristics were found in the missing patients (detailed data is shown in Additional file 1: Table S1). Finally, a total of 144 (85.2%) potential bilateral TKR patients were included. Among these, 93 (64.6%) patients selected unilateral TKR (Group 1), and 51 (35.4%) patients selected bilateral TKR (Group 2).

### Data source and definition

Data analyses were performed as a comparative cohort study, and data were collected prospectively from a single center. Demographic details including age, gender, medical history, height and weight were retrieved from hospital records. In addition, self-reported education, income and current work status before TKR surgery were recorded. The functional improvements were based on patient reported outcomes and assessed according to the WOMAC [18] and Medical Outcomes Trust Short Form-36 (SF-36) [19]. Questionnaires pertaining to socio-economic factors and patient reported outcomes were conducted during face-to-face interviews with each participant by well-trained investigators before TKR.

The WOMAC index included 24 questions divided into 3 sub-scales: pain, stiffness and function. These sub-scales were combined to produce a total measure of knee health. Each question had a visual analogue scale (VAS) for assessing functional scores (0–100-point scale; 0 best). Scores of questions in each subscale were averaged to calculate pain, stiffness, function and total scores. The SF-36 index included 36 questions, divided into 9 sub-scales: health change, physical function, role of function/physical, pain, general health, role of function/emotion, energy/fatigue, emotional well-being and social function. These sub-scales were combined as a measure of general health. Scores were transformed to produce a 0–100-point scale (100 best), and scores from each sub-scale were calculated according to a previous study [19]. Scores for physical function, role of function/physical, pain and general health were averaged to give a physical component score, and scores for role of function/emotion, energy/fatigue, and emotional well-being while social were averaged to give the mental component score. Health changes were assessed as an independent subscale.

Potential impact factors included gender, age, BMI (body mass index), education, income, current work, other bone disease, low back pain and history of disease. BMI was calculated from self-reported height and weight. Education was divided into two groups (≤ 6 years and >  6 years) because compulsory education was previously 6 years in Taiwan. To maintain privacy, income was assessed as enough or lacking. Because some patients > 65 years of age had not yet retired, we asked for current work status (without or with employment). Other bone diseases and low back pain were self-reported as with or without. History of disease was assessed using the open-ended question “Has a doctor ever diagnosed you as having any disease?” However, most diseases were rare in the present patient cohort. Only histories of cardiovascular disease (CVD), diabetes mellitus (DM) and hypertension (HTN) were analyzed because their prevalence was > 10%.

In the primary analysis, the three primary outcomes were as follows: (1) patient reported outcomes before TKR in each group, (2) patient reported outcomes after 6 months in each group and (3) changes in patient reported outcomes in each group. The first and second primary analyses used independent sample t-tests to compare the means of patient reported outcomes before and after TKR. The third primary analysis used paired t-tests to compare the means of the changes in functional outcomes in each group. It is noteworthy that assessing for change of functional outcomes after TKR within 6 months is a short follow up compared with related studies [14, 22]. However, the patients receiving unilateral TKR will continue with the second stage surgery after 6 months, so follow up after more than 6 months would be impacted by second stage surgery in patients receiving unilateral TKR. This might reduce the comparability between two groups, so this study only followed up within 6 months.

### Sample size calculation

Prior to the study, we used G*Power to perform a t-test of the difference between two independent means to calculate the required sample size [20], and effects were detected in a two-sided test with a power of (1 − β) = 80% at a significance level of 0.05. Other calculation settings were as follows: (1) the hypothetical proportion of patients selecting bilateral TKR was 40% based on clinical experience, (2) minimally clinically important differences (MCID) were at least 15 points for the WOMAC and 10 points for the SF-36 [21], and (3) the standard deviations of functional changes were approximately 20, as shown in a previous study [22]. Based on these settings, the required sample size for calculation was at least 60 subjects for the WOMAC and 133 subjects for the SF-36.

### Statistical method

All data were analyzed using the R statistical program (version 3.1.1) with the geepack package, and graphs were drawn using bear, ggplot2 and metafor packages.

### Association analysis

Categorical and continuous variables were presented as numbers (proportions) and means ± standard deviations. Differences between variables of patients in each group were tested using the Student’s t-test or the χ^2^ test where appropriate. The significance level was set at 0.05/11 = 0.0045 based on Bonferroni correction to avoid errors of multiple testing.

### Impact factor analysis

To infer the progression of a primary parameter and then apply parameter ranking to investigate which behavioral data had the highest ‘impact’ on patient reported outcomes, we used a generalized estimating equation to analyze the association between possible impact factors and changes in patient reported outcomes. Significance levels were again set at 0.05/11 = 0.0045. Accordingly, the results were presented using forest plots with 99.54% confidence intervals. Significantly associated factors in both association analyses and impact factor analyses were considered confounders and were adjusted in subsequent analyses. However, no factors met these criteria.

Patient reported outcomes before and after TKR in each group were presented as the means ± standard deviations, and changes in patient reported outcomes in each group were presented as the means with 95% confidence intervals. To investigate the association between surgery type and changes in patient reported outcomes and adjust the confounders, the generalized estimating equation was used to analyze repeated data. GEE models were adjusted by all factors (gender, age, BMI, education, income, current work, other bone disease, low back pain, history of CVD, history of DM and history of HTN). The significance level was set at 0.05. Although no potential confounders were present, fully adjusted changes in patient reported outcomes were presented for each group.

## Results

### Association analysis

Table 1 shows differences in characteristics between Groups 1 (unilateral TKR) and 2 (bilateral TKR). Group 1 comprised 75.3% females aged 70.4 ± 7.2 years old, and Group 2 comprised 90.2% females aged 70.0 ± 6.2 years old. The *p*-value for the association between gender and group was < 0.05 (*p* = 0.030) but was not significant after Bonferroni correction (significance level = 0.0045). Other factors such as age, BMI, education, income, current work, other bone disease, low back pain, history of CVD, history of DM and history of HTN were not associated with treatment selections.

**Table 1:** Demographics and patient characteristics

		Group 1	Group 2	*p*-value
		(*n* = 93)	(*n* = 51)	
Gender	Male	23 (24.7%)	5 (9.8%)	0.030
	Female	70 (75.3%)	46 (90.2%)	
Age (years)		70.4 ± 7.2	70.0 ± 6.2	0.707
BMI (kg/m^2^)		27.1 ± 3.5	27.4 ± 3.4	0.615
Education (years)	≤ 6	69 (74.2%)	44 (86.3%)	0.092
	> 6	24 (25.8%)	7 (13.7%)	
Income	Enough	86 (92.5%)	49 (96.1%)	0.393
	Lacking	7 (7.5%)	2 (3.9%)	
Current work status	Without	70 (75.3%)	39 (76.5%)	0.872
	With	23 (24.7%)	12 (23.5%)	
Other bone disease	Without	69 (74.2%)	37 (72.5%)	0.860
	With	24 (25.8%)	14 (27.5%)	
Low back pain	Without	44 (47.3%)	27 (52.9%)	0.518
	With	49 (52.7%)	24 (47.1%)	
CVD	Without	71 (76.3%)	45 (88.2%)	0.085
	With	22 (23.7%)	6 (11.8%)	
DM	Without	73 (78.5%)	40 (78.4%)	0.993
	With	20 (21.5%)	11 (21.6%)	
HTN	Without	41 (44.1%)	16 (31.4%)	0.136
	With	52 (55.9%)	35 (68.6%)	

Group 1, unilateral TKR; Group 2, bilateral TKR; CVD, history of cardiovascular disease; DM, history of diabetes mellitus; HTN, history of hypertension

Statistical significance was set at *p* < 0.0045, as described in the statistical analysis section

### Impact factor analysis

**Fig. 1:**
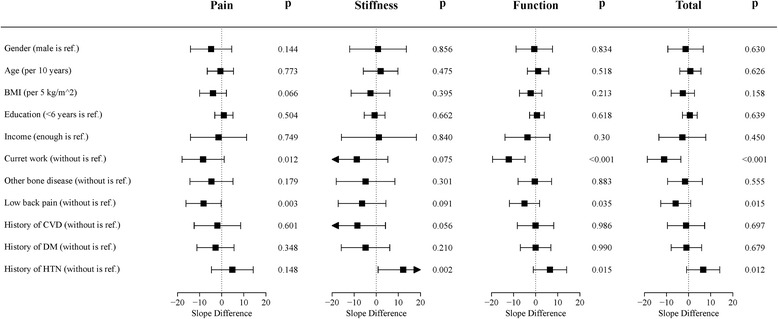
Impact and 99.54% conference interval of potential impact factors on WOMAC score. CVD: cardiovascular disease; DM: diabetes mellitus; HTN: hypertension. This forest plot included 11 potential impact factors on WOMAC score: Gender (female versus male); Age (10 years is a unit); BMI (5 kg/m^2^ is a unit); Education (> 6 versus ≤6 years); Income (lacking versus enough); Current work (with versus without); Other bone disease (with versus without); Lower back pain (with versus without); CVD (with versus without); DM (with versus without); HTN (with versus without). For each potential impact factor, generalized estimating equation analysis was used to interaction between time and impact factor

**Fig. 2:**
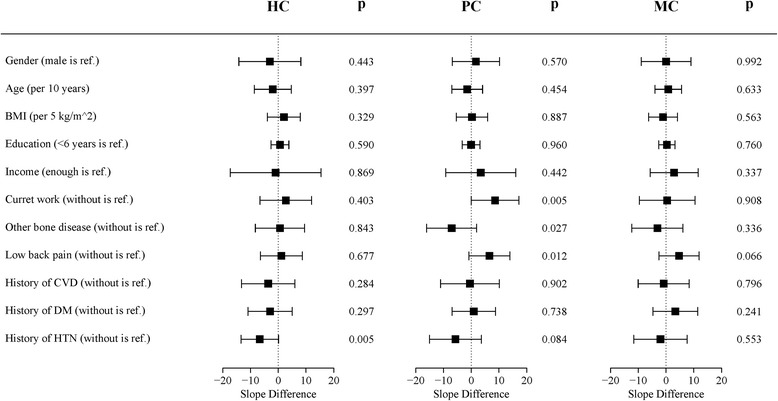
Impact and 99.54% conference interval of potential impact factors on SF-36 score. HC: health change; PC: physical component; MC: mental component; CVD: cardiovascular disease; DM: diabetes mellitus; HTN: hypertension. This forest plot included 11 potential impact factors on SF-36 score: Gender (female versus male); Age (10 years is a unit); BMI (5 kg/m^2^ is a unit); Education (> 6 versus ≤6 years); Income (lacking versus enough); Current work (with versus without); Other bone disease (with versus without); Low back pain (with versus without); CVD (with versus without); DM (with versus without); HTN (with versus without). For each potential impact factor, generalized estimating equation analysis was used to interaction between time and impact factor

Associations of potential impact factors with WOMAC and SF-36 scores (Figs. 1 and 2) indicated that patients with current work may benefit from TKR more than patients without current work (slope difference, − 11.1; 99.42% CI, from − 18.7 to − 3.5) and predominantly reflected function scores (slope difference, − 12.1; 99.42% CI, from − 19.5 to − 4.8). No other factors were associated with changes in total WOMAC scores in either univariate or multivariate models (data not shown). According to the stiffness scores, patients with a history of HTN may receive less benefit from TKR than those without a history of HTN (slope difference, 12.2; 99.42% CI, 0.9–23.5). However, history of HTN was not a significant impact factor after adjusting for current work status (*p*-value before adjustment, 0.002; *p*-value after adjustment, 0.010).

No significant impact factors were found among SF-36 scores, including in changes in health, physical components and mental component scores. However, current work status (*p* = 0.0047) and history of HTN (*p* = 0.0052) were almost significantly associated with physical component and mental component scores, respectively.

### Primary analysis

#### WOMAC

**Fig. 3:**
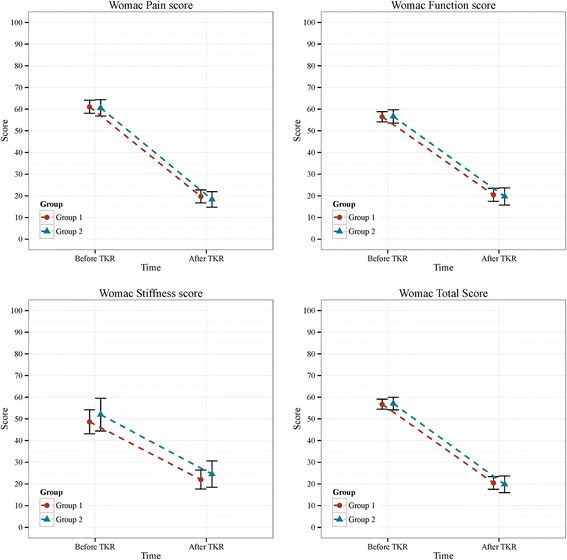
Mean WOMAC score and their 95% conference interval for each of the 2 groups. Group 1: Unilateral TKR; Group 2: Bilateral TKR

A breakdown of WOMAC scores for the 2 groups is shown in Table 2 and Fig. 3. Among Group 1 patients, mean pre-operative and 6-months total WOMAC scores were 56.8 ± 11.3 and 20.4 ± 14.2, respectively, and those among Group 2 patients were 57.1 ± 10.3 and 19.8 ± 13.8, respectively. Changes in total WOMAC scores following TKR were − 36.3 (95% CI, from − 39.3 to − 33.4) and − 37.3 (from − 41.4 to − 33.1) in Groups 1 and 2, respectively. After TKR, pain, stiffness, function and total WOMAC scores were significantly reduced in both groups, with mean changes from − 26.6 to − 41.4 and from − 27.5 to − 42.2, respectively. Both groups showed similar trends in the various sub-scales, and no significant differences in any sub-scale were observed between treatment groups before and after adjustment. The most improved sub-scale was pain, whereas only minimal improvements were observed in the stiffness sub-scale.

**Table 2:** Comparison of the WOMAC and SF-36 scores for the 2 groups

		Group 1	Group 2	95%CI	*p*-value#	*p*-value$
		(*n* = 93)	(*n* = 51)			
WOMAC						
Pain	Pre-op	61.1 ± 14.7	60.6 ± 13.5	−4.38~ 5.43	0.833	0.464
	Post-op	19.7 ± 14.6	18.3 ± 12.7	−3.43~ 6.19	0.572	0.397
	Change	−41.4 (from − 44.9 to −37.9)	− 42.2 (from − 47.2 to −37.3)	−5.14~ 6.85	0.779	0.931
Stiffness	Pre-op	48.6 ± 26.9	52.0 ± 26.8	−12.56~ 5.93	0.876	0.608
	Post-op	22.0 ± 21.2	24.5 ± 21.6	−9.87~ 4.86	0.503	0.757
	Change	−26.6 (from −31.2 to −22.0)	−27.5 (−34.1 to −20.8)	−7.19~ 8.81	0.841	0.741
Function	Pre-op	56.5 ± 11.5	56.6 ± 10.9	−4.07~ 3.71	0.926	0.821
	Post-op	20.5 ± 14.5	19.7 ± 14.1	−4.19~ 5.70	0.764	0.517
	Change	−36.0 (from − 39.0 to − 33.1)	−36.9 (from −41.1 to − 32.8)	− 4.13~ 6.01	0.716	0.631
Total	Pre-op	56.8 ± 11.3	57.1 ± 10.3	−4.06~ 3.46	0.876	0.795
	Post-op	20.4 ± 14.2	19.8 ± 13.8	−4.22~ 5.44	0.803	0.541
	Change	−36.3 (from − 39.3 to −33.4)	−37.3 (from −41.4 to − 33.1)	− 4.17~ 5.99	0.724	0.684
SF-36						
Health change	Pre-op	36.3 ± 17.9	34.8 ± 16.6	−4.52~ 7.49	0.626	0.899
	Post-op	45.2 ± 18.2	47.1 ± 17.1	−8.02~ 4.22	0.541	0.254
	Change	8.9 (5.7–12.0)	12.3 (7.6–16.9)	−8.88~ 2.12	0.226	0.166
Physical	Pre-op	28.6 ± 11.3	28.2 ± 10.8	−3.45~ 4.22	0.842	0.887
	Post-op	74.0 ± 15.4	74.0 ± 15.2	−5.25~ 5.28	0.996	0.680
	Change	45.3 (42.2–48.5)	45.7 (41.0–50.4)	−5.94~ 5.19	0.895	0.774
Mental	Pre-op	46.4 ± 15.0	48.8 ± 15.6	−7.67~ 2.78	0.357	0.254
	Post-op	77.0 ± 9.6	75.5 ± 12.1	−2.17~ 5.09	0.429	0.585
	Change	30.6 (27.6–33.7)	26.7 (22.2–31.2)	−1.41~ 9.21	0.149	0.124

Group 1, unilateral TKR; Group 2, bilateral TKR; Pre-op, pre-operative; Post-op, 6 months after surgery

#, The *p*-value

$, adjusted *p*-value; Models were adjusted by all factors (gender, age, BMI, education, income, current work, other bone disease, low back pain, history of CVD, history of DM and history of HTN)

#### SF-36

**Fig. 4:**
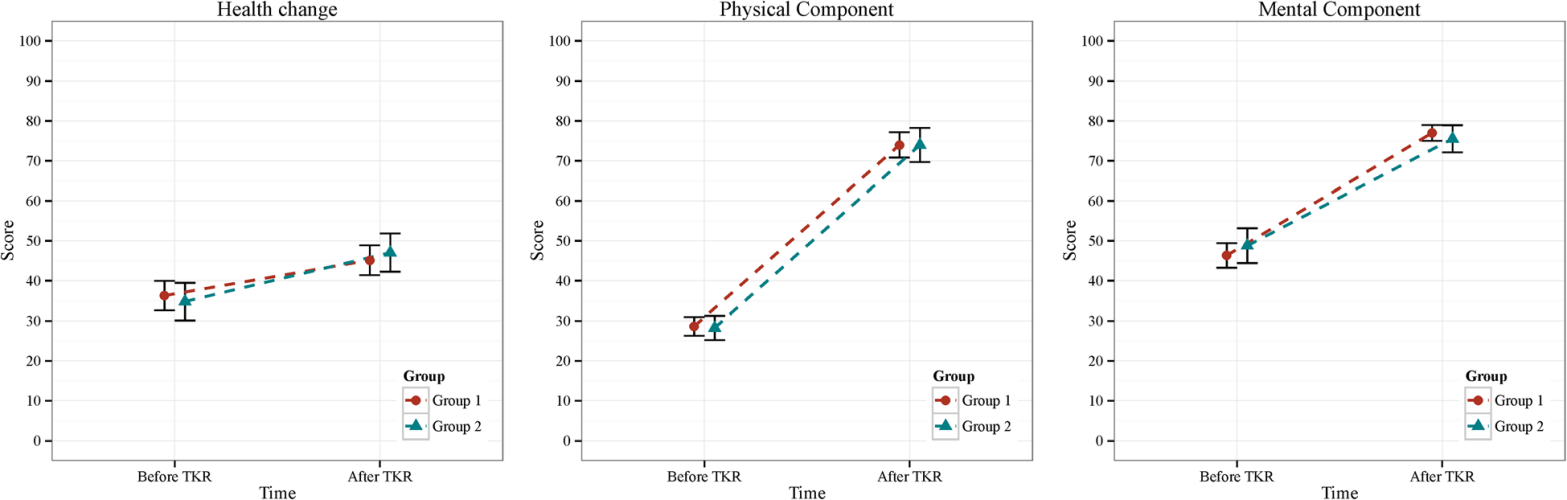
Mean SF-36 score and their 95% conference interval for each of the 2 groups. Group 1: Unilateral TKR; Group 2: Bilateral TKR

Table 2 and Fig. 4 show the breakdown of SF-36 scores among patients of the two groups. Before TKR, the mean health change, physical component and mental component scores were 36.3 ± 17.9, 28.6 ± 11.3 and 46.4 ± 15.0, respectively, in Group 1 and 34.8 ± 17.9, 28.2 ± 10.8 and 48.8 ± 15.6, respectively, in Group 2. After TKR, the mean health change, physical component and mental component scores changed to 45.2 ± 18.2, 74.0 ± 15.4 and 77.0 ± 9.6, respectively, in Group 1 and 47.1 ± 17.1, 74.0 ± 15.2 and 75.5 ± 12.1, respectively, in Group 2.

Health change, physical component and mental component SF-36 scores were significantly increased after TKR by 8.9 (95% CI, 5.7–12.0), 45.3 (95% CI, 42.2–48.5) and 30.6 (95% CI, 27.6–33.7), respectively, among Group 1 patients and 12.3 (95% CI, 7.6–16.9), 45.7 (95% CI, 41.0–50.4) and 26.7 (95% CI, 22.2–31.2), respectively, among Group 2 patients. In agreement with the WOMAC scores, trends in sub-scales of SF-36 scores were similar in the two treatment groups, and no significant differences were identified before or after adjustment. Moreover, the greatest improvement following TKR was in the physical component sub-scale.

## Discussion

In the present study, no differences in investigated characteristics were found between patients receiving unilateral and bilateral TKR. In addition, all patients had substantial functional improvements following surgery. Although no differences in functional improvement sub-scales were found between treatment groups, current work status influenced the perceived benefits of the interventions.

This study population had similar characteristics to those of other studies [12–14, 22], with a mean age of approximately 70 years and more females than males. The proportion of patients who selected bilateral TKR was 36.6% in a previous study [14] and 35.4% in the present study. In addition, the range of mean WOMAC scores at baseline was 50–60 in both studies, and the ranges of mean physical component and mental component SF-36 scores at baseline were 20–30 and 40–50, respectively, indicating that the present cohort is representative.

It is widely recognized that TKR improves quality of life for OA patients [2]. In agreement, the present functional improvements in patients receiving unilateral and bilateral TKR were both statistically and clinically significant and were greater than MCID [21].

Previous studies also report significant differences in functional improvements between patients receiving unilateral and bilateral TKR, and the present patient groups had differences in health insurance because of the high rate of private health insurance [14]. In particular, patients receiving bilateral TKR suffered from increased discomfort in the short-term compared with those receiving unilateral TKR [6], but this might not impact the functional outcome after rehabilitation. Nonetheless, other factors in addition to functional improvements may require consideration during decision making for bilateral or unilateral TKR. Functional outcomes might not be the only factor to decide the modes of TKR: safety and financial matters are also variables to consider when deciding between bilateral TKA and staged procedures. Although it might be disputed, some evidence exists that the risk of complications following simultaneous bilateral TKR is not increased compared with that following unilateral TKR [10, 23, 24]. In addition, simultaneous bilateral TKR is reportedly more cost effective than staged bilateral TKR, although the ensuing functional improvements do not differ [11]. The above evidence needs to be considered in the decision surrounding the method of TKR, and the functional outcomes are also a critical factor in decision making.

Among the present patients, current work status was a significant impact factor, with greater improvements in WOMAC function scores following TKR in working patients. This observation may reflect greater perceptions of functional improvement among working patients who use their knees more often than non-working patients. Moreover, working patients may benefit because they will miss less work. Accordingly, current work status can be considered a novel impact factor for functional improvement following TKR. Moreover, history of HTN was significantly predictive of functional improvements following TKR but was not an independent risk factor after adjusting for work status, reflecting the prevalence of hypertension among retired patients. In agreement with the present study, a previous Saudi Arabian study of potential impact factors for the effect of TKR showed no association between gender and functional improvement [25]. Moreover, a study from the United Kingdom showed no association of BMI with functional improvements following TKR [22].

This study had 3 limitations. First, although the present study was not an authentic randomized controlled trial, it had an important advantage in the homogeneity between patients receiving bilateral or unilateral TKR. Previous studies of this issue encountered some challenges, namely, patients who selected bilateral TKR had a higher proportion of private health insurance [14]. The differences in financial capability might be a potential confounder of this issue. Thus, the absence of economic influences, because the cost of TKR was covered by NHI, on patient selections of surgical procedures adds credibility. Moreover, Hooper and his co-workers noted some differences between patients receiving unilateral and bilateral TKR in the New Zealand National Joint Registry [24]. This real-world observation showed that age, pain, and activities of daily living might impact the selection of surgery. However, this study attempted to reduce the potential confounders, such as to exclude patients with a high risk of complications. These efforts allowed some patients who selected to carry out unilateral TKR to be excluded and reduced the potential confounders in our result. Finally, we also observed significant baseline differences between the two groups. Thus, we considered the homogeneity between two groups in our study to be acceptable. Second, assessments of outcome were based on a structured questionnaire, and self-reporting may have led to misclassification. Therefore, highly trained interviewers regularly re-standardized analyses. In addition, patients were well informed prior to interviews. Third, this was not long-term research, so there was not much time to follow the patients. We cannot completely evaluate the functional difference between the 12 month follow-up of the one-stage, simultaneous operation group and the 6 month follow-up of the second operation of the two-stage operation group.

## Conclusions

In conclusion, no differences in functional improvements were identified between patients who selected unilateral or bilateral TKR, resulting in no recommendation for one or the other procedure. It was noteworthy that we excluded the patients with serious co-morbidities, so this conclusion might be not extrapolated to them. Nonetheless, it remains critical that physicians inform patients of the differences in short-term discomfort, cost effectiveness, morbidity and mortality between the procedures. Elderly patients, or those with serious co-morbidities, might be not appropriate for bilateral TKR because they might be at increased risk for perioperative complications. Finally, the present analyses identified current work status as a novel impact factor, as those patients might be more sensitive and miss less time from work. Future studies are required to confirm this observation.

## Additional file

Additional file 1: **Table S1.** Characteristics between patients who loss to follow-up or not. (DOCX 35 kb)

## Abbreviations

BMI: Body mass index
CVD: Cardiovascular disease
DM: Diabetes mellitus
HTN: Hypertension
MCID: Minimally clinically important differences
NHI: National Health Insurance
OA: Osteoarthritis
SF-36: Medical Outcomes Trust Short Form-36
TKR: Total knee replacement
VAS: Visual analogue scale
WOMAC: Western Ontario and McMaster University osteoarthritis index
