# Supplementary material for: No difference in the functional improvements between unilateral and bilateral total knee replacements
Source: BMC Musculoskelet Disord. 2018 Mar 21;19:87. doi: 10.1186/s12891-018-2006-x (PMC5861654; doi:10.1186/s12891-018-2006-x)
Supplement: Supplementary file 1 — Table S1. Characteristics between patients who loss to follow-up or not. (DOCX 35 kb) [file 12891_2018_2006_MOESM1_ESM.docx]

**Table S1 Characteristics between patients who were lost to follow-up or not.**

|  |  | **Complete follow-up** | **Loss to follow-up** | p-value |
| --- | --- | --- | --- | --- |
|  |  | (n = 144) | (n = 25) |  |
| **TKR mode** | Unilateral | 93 (64.6%) | 16 (64.0%) | 0.955 |
|  | Simultaneous | 51 (35.4%) | 9 (36.0%) |  |
| **Gender** | Male | 28 (19.4%) | 9 (36.0%) | 0.065 |
|  | Female | 116 (80.6%) | 16 (64.0%) |  |
| **Age (years)** |  | 70.3 ± 6.8 | 73.1 ± 6.6 | 0.058 |
| **BMI (kg/m^2^)** |  | 27.2 ± 3.5 | 25.7 ± 3.6 | 0.051 |
| **Education (years)** | ≤ 6 | 113 (78.5%) | 20 (80.0%) | 0.863 |
|  | > 6 | 31 (21.5%) | 5 (20.0%) |  |
| **Income** | Enough | 135 (93.7%) | 24 (96.0%) | 0.660 |
|  | Lacking | 9 (6.3%) | 1 (4.0%) |  |
| **Current work status** | Without | 109 (75.7%) | 19 (76.0%) | 0.974 |
|  | With | 35 (24.3%) | 6 (24.0%) |  |
| **Other bone disease** | Without | 106 (73.6%) | 19 (76.0%) | 0.802 |
|  | With | 38 (26.4%) | 6 (24.0%) |  |
| **Low back pain** | Without | 71 (49.3%) | 10 (40.0%) | 0.390 |
|  | With | 73 (50.7%) | 15 (60.0%) |  |
| **CVD** | Without | 116 (80.6%) | 20 (80.0%) | 0.948 |
|  | With | 28 (19.4%) | 5 (20.0%) |  |
| **DM** | Without | 103 (78.5%) | 19 (76.0%) | 0.925 |
|  | With | 31 (21.5%) | 6 (24.0%) |  |
| **HTN** | Without | 57 (39.6%) | 9 (36.0%) | 0.735 |
|  | With | 87 (60.4%) | 16 (64.0%) |  |

CVD, cardiovascular disease; DM, history of diabetes mellitus; HTN, history of hypertension
